# A systematic literature review of herpes zoster incidence worldwide

**DOI:** 10.1080/21645515.2020.1847582

**Published:** 2021-03-02

**Authors:** Désirée van Oorschot, Hilde Vroling, Eveline Bunge, John Diaz-Decaro, Desmond Curran, Barbara Yawn

**Affiliations:** a Health Economics, GSK, Wavre, Belgium; b Pallas Health Research and Consultancy, Rotterdam, The Netherlands; c Vaccine, GSK, Rockville, MD, USA; d Department of Family and Community Health, University of Minnesota, Minneapolis, MN, USA

**Keywords:** Incidence, herpes zoster, shingles, adults, vaccination, epidemiology, review

## Abstract

We conducted a systematic review to characterize the incidence rate of herpes zoster (HZ) in the general population, specifically in individuals ≥50 years of age. A total of 69 publications were included in the review. We found a cumulative incidence of HZ ranging from 2.9–19.5 cases per 1,000 population and an incidence rate of HZ ranging from 5.23–10.9 cases per 1,000 person-years. The cumulative incidence (3.22–11.2 versus 2.44–8.0 cases per 1,000 population) and incidence rates (6.05–12.8 versus 4.30–8.5 cases per 1,000 person-years) were higher in females than males. Studies revealed a trend of increasing incidence of HZ with increasing age and over time. Variations in incidence estimates can be attributed to the various study designs, case ascertainments, age distributions of the population and year of the study. HZ is associated with a substantial disease burden and is expected to increase due to population aging.

## Introduction

Primary infection with the varicella zoster virus (VZV) often leads to acute varicella or chickenpox, typically in childhood. After recovery from chickenpox, the virus remains dormant in the dorsal root ganglia.^1^ Age-related decline in immunity or an immunosuppressed condition may lead to the reactivation of VZV causing herpes zoster (HZ), also called shingles. HZ is distinguished by a painful or pruritic, commonly unilateral, blistering rash. Although pain may persist for much longer, the average duration of the HZ rash ranges from 7 to 10 days, with the skin healing completely within approximately 2 to 4 weeks.^2^

The pain associated with HZ has been described as aching, burning, stabbing, or shock-like. Individuals with HZ may also experience altered sensitivity to touch, pain provoked by trivial stimuli, and unbearable itching.^3^ The median duration of pain is approximately 32.5 days (the mean duration is 45 days).^3^ Postherpetic neuralgia (PHN) is frequently defined as pain persisting for at least 3 months after rash onset, and occurs in 5% to 30% of patients.^4^ Pain associated with PHN can disrupt all aspects of daily life and patients with PHN may experience depression, reduced quality of life, and social withdrawal.^2^ Other complications associated with HZ include stroke or other cardiovascular events, neurological sequelae, palsy and gastrointestinal ailments.^5^ Severe cases of the above complications often require hospitalization.^5^

The lifetime risk of HZ disease without vaccination ranges between 20% and 30%.^6,7^ Gender, ethnicity, family history, and comorbidities such as systemic lupus erythematosus, asthma, diabetes mellitus and chronic obstructive pulmonary disease are risk factors for HZ.^8^ An increase in age leads to higher incidence and severity of HZ disease, especially after the age of 50 years, due to age-related decline in immunity.^9^ Considering the significance of age as a risk factor, the increasing life expectancy in the general population may considerably increase HZ annual cases and disease burden.^10^ It is becoming crucial for healthcare professionals and health policy-makers to be informed of the latest evidence on the disease burden of HZ. Findings from a previous review conducted in 2014 provides a comprehensive overview of HZ as a significant global health burden.^4^ To our knowledge, there are no reviews summarizing the evidence from more recently conducted epidemiology and burden of disease studies.

The objective of this review is to provide an up-to-date evidence base on the incidence of HZ. Specifically, this review aims to summarize the incidence rates of HZ in the general population with a focus on individuals ≥50 years of age (YOA). In addition, when available in the literature, the incidence of HZ by risk factors such as gender, age, ethnicity and immunocompetence is described. Trends of HZ incidence stratified by different geographical regions and over time are also presented.

## Methods

We performed a systematic review of the literature according to guidelines specified in the Cochrane Handbook for Systematic Reviews of Interventions^11^ and Preferred Reporting Items for Systematic Literature Reviews and Meta-Analyses (PRISMA)^12,13^ to obtain relevant information using a reproducible, robust and transparent methodology.

### Search sources and strategy

We searched the following online databases: PubMed, Embase, and the Virtual Health Library (VHL) including the Latin American & Caribbean Health Sciences Literature (Lilacs) database. The search strategy was developed using both indexed terms and terms described in the title or abstract. Search terms for the different databases were combined using Boolean operators. Details of the search strategy are provided in Supplementary Table 1. All searches were restricted by publication date from 1 January 2002 onwards and were conducted on 7 December 2018.

### Article selection and quality control

Publications identified from the searches were screened in three phases using the inclusion and exclusion criteria provided in Supplementary Table 2.

In the first phase, publications were screened based on the titles and abstracts. All titles and abstracts were screened in duplicate by two independent researchers (HV, EB). The results were compared, and deviations were discussed. In the second phase, the first 10% of eligible full-text publications were checked for relevancy in duplicate by two independent researchers (HV, EB). The results were compared and discussed early in the process to minimize the differences between both researchers with regards to the full-text publications screened in duplicate.

The process of selection of publications was registered in an EndNote library by one of the researchers. The full-text selection was documented per article with reason of exclusion in an Excel file to ensure that a clear overview of all selection steps across all phases was maintained, and reproducibility of the results was assured.

### Data extraction, quality assessment and descriptive analyses

After the eligible publications were identified for this review, one researcher (HV) extracted the relevant data from these publications into an Excel database. A reviewer (EB) quality checked the extracted data. Data extraction parameters were established *a priori* and included publication details, country, study characteristics (design, time period, setting), population characteristics (inclusion and exclusion criteria, sample size, age groups, gender, ethnicity, underlying immunocompromising conditions), methodology (case detection, case definition, type of patients and incidence denominator), incidence of HZ (per person and per person-years separately) by gender, age, ethnicity, year and case definition and information to assess the quality of the study.

There are several well-known and validated checklists available to assess the quality of publications with “classical” study designs (e.g., cohort, case-control, randomized controlled trial, etc.). However, there are no formal validated checklists which focus on incidence studies that are typically designed as surveillance studies. We defined three questions in order to assess the quality of the included HZ incidence studies (Supplementary Table 3).

In this paper, we provide a descriptive overview of the incidence of HZ in the general population ≥50 YOA. Incidence (number of HZ cases per 1,000 population hereafter referred to as cumulative incidence or as number of HZ cases per 1,000 person-years hereafter referred to as incidence rate) is presented for the overall population and for the general population stratified by gender, age, ethnicity, study year and case definition. Where incidence was expressed as a cumulative, we transformed the data from a percentage (i.e., per population) to an incidence rate assuming an exponential distribution (i.e., s(t) = 1-exp(-*ƴt*) where *ƴ* is the incidence rate and *t* is time, assumed to be equal to 1.^14^ This transformation facilitated the presentation of trends in HZ incidence.

## Results

### Included studies

**Figure 1.**
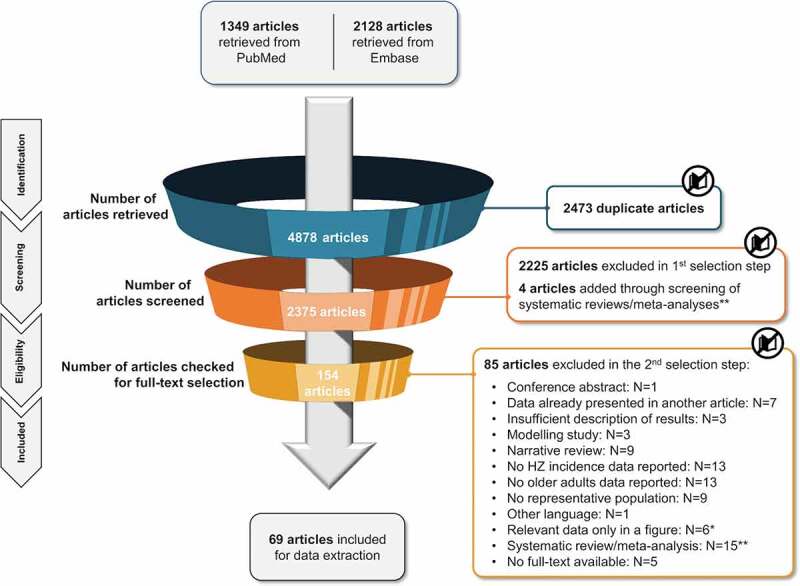
PRISMA diagram

A total of 4,848 publications were identified from the databases. After the removal of duplicates, titles and abstracts of 2,375 publications were screened for eligibility based on the pre-specified inclusion and exclusion criteria (Supplementary Table 2). After excluding 2,225 publications based on title and abstract screening, 150 full-text publications were assessed for full-text eligibility using the same criteria. A total of 69 publications were included in the review Figure 1.

*The authors were contacted to retrieve the underlying data (numerators and denominators) of these figures.**Systematic reviews/meta-analyses were checked for possibly missed relevant original articles.HZ, herpes zoster; N, number; PRISMA, Preferred Reporting Items for Systematic Reviews and Meta-Analyses

### Study characteristics

**Figure 2.**
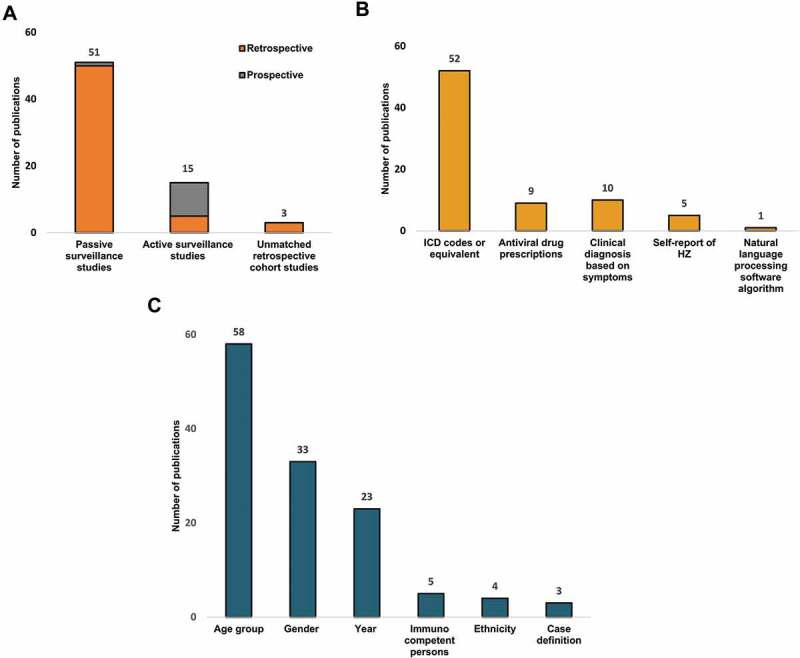
Distribution of publications (N = 69) by (A) Study design, (B) Case identification method, (C) Incidence data stratification. The total number of studies add up to 77 studies as 8 studies utilized more than one method to detect HZ (see Table 1)

HZ, herpes zoster; ICD, International Classification of Diseases

The characteristics of the 69 individual studies are provided in Table 1. The majority of studies were conducted in the United States of America (USA; n = 14),^6,15–27^ followed by Canada (n = 6),^28–33^ the United Kingdom (UK; n = 6),^34–39^ Germany (n = 5),^40–44^ Japan (n = 5),^45–49^ the Netherlands (n = 5),^36,50–53^ Spain (n=5),^54–58^ Taiwan (n = 4),^59–62^ Australia (n = 3),^63–65^ China (n = 3),^66–68^ Italy (n = 3),^69–71^ France (n = 2),^72,73^ New Zealand(n = 2),^74,75^ Sweden (n = 2),^76,77^ Denmark (n = 1),^78^ Israel (n = 1),^79^ Norway (n = 1),^80^ Poland (n = 1),^81^ and South Korea (n = 1).^82^ One study presented data for both the Netherlands and the UK separately;^36^ this study is captured within the individual countries Figure 2; Table 1.

**Table 1.** Characteristics of included studies (N = 69)

Author, year	Country	Study design	Study period	Setting	Age (years)	Case definition	Patient type	Type of incidence data reported	Stratifications
Liu, 2015^63^	Australia	Prospective passive surveillance study	1 January 2006–31 December 2009	The 45 and Up Study in New South Wales	≥45	Drug prescription or ICD codes (inpatients only)	Inpatients and outpatients	Incidence rate	Age
MacIntyre, 2015^64^	Australia	Retrospective passive surveillance study	1 July 2006–31 March 2013	6,302 GPs captured in the Bettering the Evaluation of Care and Health database, and the (Repatriation) Pharmaceutical Benefits Scheme database	≥50	ICPC codes or drug prescription	Outpatients	Cumulative incidence	Age, case definition (GP visits or antiviral prescriptions)
Stein, 2009^65^	Australia	Retrospective passive surveillance study	1 April 2000–30 September 2006	6,460 GPs captured in the Bettering the Evaluation of Care and Health database, and the (Repatriation) Pharmaceutical Benefits Scheme database	≥50	ICPC codes or drug prescription	Outpatients	Cumulative incidence	Age, case definition (GP visits or antiviral prescriptions)
McDonald, 2017^30^	Canada	Retrospective passive surveillance study	1 November 2009–31 October 2015	Alberta Health Care Insurance Plan Registry, an Albertan health insurance	≥50	ICD codes	Inpatients and outpatients	Incidence rate	Age, gender
Marra, 2016^29^	Canada	Retrospective passive surveillance study	1 January 1997–31 December 2012	Population-DataBC® Medical Services Plan and Discharge Abstract Database, linked to the outpatient prescription database PharmaNet; British Columbia	≥50	ICD codes	Inpatients and outpatients	Cumulative incidence	Age, year
Russel, 2014^31^	Canada	Retrospective passive surveillance study	1 January 1994–31 December 2010	Alberta’s universal, publicly funded health-care insurance system databases	≥50	ICD codes	Inpatients and outpatients	Cumulative incidence	Age, year
Tanuseputro, 2011^33^	Canada	Retrospective passive surveillance study	1 April 1992–31 March 2010	Canadian Institute of Health Information Discharge Abstract Database (Ontario) and Ontario Health Insurance Plan	≥50	ICD codes	Inpatients and outpatients	Cumulative incidence	Age, year
Russel, 2007^32^	Canada	Retrospective passive surveillance study	1 January 1990–31 December 2002	Hospital Morbidity Inpatient database and the Alberta Health Care Insurance Plan Registry; Alberta Province	≥50	ICD codes	Inpatients and outpatients	Cumulative incidence	Age, gender, year
Edgar, 2007^28^	Canada	Retrospective passive surveillance study	1 January 1994–31 December 2003	British Columbia Ministry of Health Medical Services Plan database (physician billing data)	≥65	ICD codes	Outpatients	Cumulative incidence	-
Lu, 2018^67^	China	Retrospective active surveillance study	December 2012 – March 2013	52 communities/villages in three districts of Beijing (Xicheng, Changping and Miyun)	≥50	Self-report	Outpatients	Cumulative incidence	Age, gender
Li, 2016^66^	China	Retrospective active surveillance study	May 2013 – May 2014	One rural township each in Jiangsu, Jiangxi, Heilongjiang and Hebei and one community from Shanghai	≥50	Self-report	Inpatients and outpatients	Cumulative incidence	Age, gender, year
Zhu, 2015^68^	China	Retrospective active surveillance study	28 October 2013 – NR	34 of the 126 counties/districts in Guangdong Province, selected using random sampling	≥50	Self-report	Outpatients	Cumulative incidence	Year
Schmidt, 2017^78^	Denmark	Retrospective passive surveillance study	1 January 1997–31 December 2013	Danish National Prescription Registry and Danish National Patient Registry	≥50	Drug prescription ICD codes (inpatients only)	Inpatients and outpatients	Cumulative incidence	Age, gender
Amirthalingam, 2018^34^	England	Retrospective unmatched cohort study	1 October 2005–30 September 2016	164 Royal College of General Practitioners – Research and Surveillance Centre practices across England	60–89	READ codes	Outpatients	Incidence rate	Age, gender, year
Jain, 2018^38^	England	Retrospective passive surveillance study	1 September 2003–31 August 2013	385 Clinical Practice Research Datalink practices across England	≥65	CPRD codes ICD codes (inpatients only)	Inpatients and outpatients	Incidence rate	Age, ethnicity, gender
Mick, 2010^73^	France	Prospective active surveillance study	1 January 2005–31 December 2005	225 GPs, 36 dermatologists, 15 neurologists and 5 physicians in pain clinics from a random sample of these physician types	≥50	Clinical	Outpatients	Cumulative incidence	Age, gender
Gonzalez Chiappe, 2010^72^	France	Retrospective passive surveillance study	1 January 2000–31 December 2008	~1200 GPs reporting to the French general practitioners’ Sentinelles electronic surveillance network	≥45	Clinical	Outpatients	Cumulative incidence	Age
Schmidt-Ott, 2018^42^	Germany	Prospective active surveillance study	November 2010 – December 2014	GPs, dermatologists and ophthalmologists in 3 German regions (Fulda, Leverkusen and Marl)	≥50	Clinical	Outpatients	Incidence rate	Age, gender
Hillebrand, 2015^40^	Germany	Retrospective passive surveillance study	1 January 2005–31 December 2009	German Pharmacoepidemiological Research Database, a national database	≥50	ICD codes	Inpatients and outpatients	Incidence rate	Year
Ultsch, 2013^43^	Germany	Retrospective passive surveillance study	1 January 2005–31 December 2008	German Statutory Health Insurance System Allgemeine Ortskrankenkasse and Regional Association of SHI-Accredited Physicians (KV) in Hessen	≥50	ICD codes	Inpatients and outpatients	Incidence rate	Age
Ultsch, 2011^44^	Germany	Retrospective passive surveillance study	1 January 2007–31 December 2008	Association of Statutory Health Insurance Physicians database	≥50	ICD codes	Outpatients	Cumulative incidence	Age, gender, year
Schiffner-Rohe, 2010^41^	Germany	Retrospective passive surveillance study	1 January 2003–31 December 2004	An insurance database (Allgemeine Ortskrankenkasse Hessen/KV Hessen)	≥50	ICD codes	Inpatients and outpatients	Cumulative incidence	Age, gender, population (general population and IC only)
Alicino, 2017^69^	Italy	Retrospective passive surveillance study	1 January 2013–31 December 2015	56 GPs in Liguria, Puglia, Toscana and Veneto	≥50	ICD codes or drug prescription	Outpatients	Incidence rate	Age, gender
Gialloreti, 2010^71^	Italy	Retrospective passive surveillance study	1 January 2003–31 December 2005	342 GPs reporting in the Health Search Database of the Società Italiana Medici Generici, from Northern, Central and Southern Italy	≥50	ICD codes	Outpatients	Cumulative incidence	Age, population (general population and IC only)
Di Legami, 2007^70^	Italy	Prospective active surveillance study	1 January 2004–31 December 2004	All 24 GPs working in Torino and Cuorgnè, Piemonte	≥45	Clinical	Outpatients	Cumulative incidence	Age
Weitzman, 2013^79^	Israel	Retrospective passive surveillance study	1 January 2006–30 September 2010	Maccabi Healthcare Services database	≥45	ICD codes	Inpatients and outpatients	Incidence rate	Age
Toyama, 2018^49^	Japan	Prospective active surveillance study	1 January 1997–31 December 2017	33 dermatology clinics and dermatology departments of 10 flagship general hospitals associated with the Miyazaki Dermatologist Society, in Miyazaki Prefecture	≥60	Clinical	Outpatients	Cumulative incidence	Year
Imafuku, 2018^45^	Japan	Retrospective passive surveillance study	1 January 2005–31 December 2014	Japan Medical Data Center-Claims Database, a national health insurance database	50–74	ICD codes and drug prescription	Inpatients and outpatients	Incidence rate	Age, gender
Shiraki, 2017^46^	Japan	Prospective active surveillance study	1 June 2009–30 November 2015	36 dermatology clinics and dermatology departments of 7 flagship general hospitals belonging to the Miyazaki Dermatologist Society, Miyazaki Prefecture	70–79	Clinical	Outpatients	Cumulative incidence	Gender
Takao, 2015^47^	Japan	Prospective active surveillance study	1 December 2008–30 November 2012	Shozu County, Kagawa Prefecture, Japan	≥50	Clinical	Inpatients and outpatients	Incidence rate	Age, gender
Toyama, 2009^48^	Japan	Prospective active surveillance study	1 January 1997–31 December 2006	39 dermatology clinics and dermatology departments of 7 flagship general hospitals associated with the Miyazaki Dermatologist Society, in Miyazaki Prefecture	≥50	Clinical	Outpatients	Cumulative incidence	Age, gender
Pierik, 2012^53^	NL	Retrospective passive surveillance study	1 January 2004–31 December 2008	ZorgGroep Almere, a database of 22 GPs in Almere	≥60	ICPC codes	Outpatients	Cumulative incidence	Age
Opstelten, 2006^52^	NL	Prospective active surveillance study	1 January – 31 December 2001	Second Dutch National Survey of General Practice; 186 GPs in 90 practices nationwide	≥45	ICPC codes	Outpatients	Cumulative incidence	Age, gender (no both genders data)
Opstelten, 2005^51^	NL	Prospective active surveillance study	1 January – 31 December 2001	Second Dutch National Survey of General Practice; 186 GPs in 90 practices nationwide	≥65	ICPC codes	Outpatients	Cumulative incidence	-
Opstelten, 2002^50^	NL	Retrospective passive surveillance study	1 August 1994–31 July 1999	Huisartsen Netwerk Utrecht, a general practice research database in the province of Utrecht; 22 GPs in 6 locations	≥45	ICPC codes	Outpatients	Incidence rate	Age
Rimseliene, 2016^80^	Norway	Retrospective passive surveillance study	2008–2012	Norwegian Health Economics Administration database	≥70	ICPC codes	Outpatients	Cumulative incidence	-
Turner, 2018^75^	New Zealand	Retrospective passive surveillance study	1 January 2005–31 December 2015	39 consenting general practices from two primary health organizations in lower North Island	≥50	natural language processing software algorithm	Outpatients	Incidence rate	Age, year
Reid, 2014^74^	New Zealand	Retrospective passive surveillance study	1 January 2009–31 December 2013	A large group practice in Lower Hutt	≥51	Coding (NR) or drug prescription	Outpatients	Cumulative incidence	Age, gender
Albrecht, 2015^81^	Poland	Retrospective passive surveillance study	2013	Świętokrzyskie Province Division of the National Health Fund	≥50	ICD codes	Inpatients and outpatients	Cumulative incidence	-
Kim, 2014^82^	South Korea	Retrospective passive surveillance study	1 January 2011–31 December 2011	National Health Insurance Service database	≥50	ICD codes	Inpatients and outpatients	Cumulative incidence	Age
Muñoz-Quiles, 2017^58^	Spain	Retrospective passive surveillance study	1 January 2009–31 December 2014	SIA (not defined) database and Hospitalization Minimum Data Set, Valencian Region	≥50	ICD codes	Inpatients and outpatients	Incidence rate	-
Esteban-Vasallo, 2014^55^	Spain	Retrospective passive surveillance study	1 January 2005–31 December 2012	Madrid regional public health system	≥45	ICPC codes	Outpatients	Cumulative incidence	Age, gender, year
Morant-Talamante, 2013^57^	Spain	Retrospective passive surveillance study	1 January 2007–20 December 2010	Abucasis electronic medical database and the Hospital Data Surveillance System, in the Valencian community	≥50	ICD codes	Inpatients and outpatients	Incidence rate	Age, gender, year
Cebrian-Cuenca, 2010^54^	Spain	Prospective active surveillance study	1 December 2006–30 November 2007	24 GP offices of the public healthcare system of the Autonomous Community of Valencia	≥50	Clinical	Outpatients	Cumulative incidence	Age
Garcia-Cenoz, 2008^56^	Spain	Retrospective passive surveillance study	1 January 2005–31 December 2006	“La base de datos de la historia clinica informatizada de atencion primaria”, Navarra	≥50	ICPC codes	Outpatients	Cumulative incidence	Age
Sundström, 2015^77^	Sweden	Retrospective passive surveillance study	January 2008 – December 2010	Västra Götaland County	≥50	ICD codes	Inpatients and outpatients	Incidence rate	Age, gender, year
Nilsson, 2015^76^	Sweden	Retrospective passive surveillance study	1 January – 31 December 2011	Swedish National Pharmacy Register	≥50	Drug prescription	Inpatients and outpatients	Cumulative incidence	Age
Lu, 2018^62^	Taiwan	Retrospective passive surveillance study	1 January 2004–31 December 2008	NHIRD, a national health insurance database	≥50	ICD codes	Inpatients and outpatients	Cumulative incidence	Age, year
Chao, 2011^59^	Taiwan	Retrospective passive surveillance study	1 January 2000–31 December 2008	National Health Insurance Research Database	≥50	ICD codes	Outpatients	Cumulative incidence	Age, year
Lin, 2010^62^	Taiwan	Retrospective passive surveillance study	1 January 2000–31 December 2005	National Health Insurance Research Database	≥50	ICD codes	Inpatients and outpatients	Cumulative incidence	Age
Jih, 2009^60^	Taiwan	Retrospective passive surveillance study	1 January 2000–31 December 2006	National Health Insurance Research Database	>80	ICD codes	Inpatients and outpatients	Incidence rate	-
Walker, 2018^39^	UK	Retrospective passive surveillance study	1 September 2013–31 August 2016	Clinical Practice Research Datalink practices across the UK (number of practices NR)	68–79	READ codes ICD codes (inpatients only)	Inpatients and outpatients	Incidence rate	Gender
Gauthier, 2009^37^	UK	Retrospective passive surveillance study	1 January 2000–31 March 2006	603 General Practice Research Datalink practices, the UK	≥50	GPRD codes	Outpatients	Incidence rate	Age, gender
Fleming, 2004^36^	UK and NL	Retrospective passive surveillance study	1 January 1994–31 December 2001	Weekly Returns Service of the Royal College of General Practitioners in England and Wales and Dutch Sentinel practice network (number of GPs NR)	≥45	Clinical	Outpatients	Cumulative incidence	Age, gender (no “both genders” data)
Brisson, 2003^35^	UK	Retrospective passive surveillance study	1 January 1991–31 December 2000	69 Royal College of General Practitioners practices across England and Wales and participating GPs in the National Survey of Morbidity in General Practice database	≥45	ICD codes	Inpatients and outpatients	Cumulative incidence	Age
Harpaz, 2018^27^	USA	Retrospective passive surveillance study	1 January 1998–31 December 2016	Two Medstat MarketScan databases: Commercial Claims and Encounters, and Medicare Supplemental and Coordination of Benefits	≥45	ICD codes	Outpatients	Cumulative incidence	Age, year
Kawai, 2016^19^	USA	Retrospective passive surveillance study	1 January 1980–31 December 2007	Rochester Epidemiology Project, conducted in Olmsted County, Minnesota	≥50	ICD codes	Inpatients and outpatients	Incidence rate	Age, year
Johnson, 2015^18^	USA	Retrospective passive surveillance study	1 January 2011–31 December 2011	Two Medstat MarketScan databases: Commercial Claims and Encounters, and Medicare Supplemental and Coordination of Benefits	≥50	ICD codes	Inpatients and outpatients	Incidence rate	Age, gender
Chen, 2014^16^	USA	Retrospective passive surveillance study	1 January 2005–31 December 2009	Commercially insured, Medicare and Medicaid administrative medical and pharmacy claims databases	≥50	ICD codes	Inpatients and outpatients	Incidence rate	Age
Krishnarajah, 2014^20^	USA	Retrospective passive surveillance study	1 January 2006–31 December 2010	MarketScan Medicaid database	50–64	ICD codes	Inpatients and outpatients	Cumulative incidence	Age, gender, year
Suaya, 2014^24^	USA	Unmatched retrospective cohort study	1 January 2005–31 December 2009	Three MarketScan databases: Commercial, Medicare and Medicaid databases	≥50	ICD codes	Inpatients and outpatients	Incidence rate	Age, population (general population and IC only)
Hales, 2013^17^	USA	Retrospective passive surveillance study	1 January 1992–31 December 2010	Medicare, a national health insurance program	>65	ICD codes	Inpatients and outpatients	Incidence rate	Age, ethnicity, gender, year
Langan, 2013^21^	USA	Unmatched retrospective cohort study	1 January 2007–31 December 2009	Medicare, a national health insurance program	≥65	ICD codes and/or drug prescription	Inpatients and outpatients	Incidence rate	Age, ethnicity, gender, population (general population and IC only), case definition (with or without antiviral prescriptions)
Leung, 2011^22^	USA	Retrospective passive surveillance study	1 January 1993–31 December 2006	Two Medstat MarketScan databases: Commercial Claims and Encounters, and Medicare Supplemental and Coordination of Benefits	≥45	ICD codes	Outpatients	Cumulative incidence	Age, gender, year
Chaves, 2007^15^	USA	Retrospective active surveillance study	2 February 2004–27 May 2004	National Adult Immunization Survey	≥65	Self-report	Outpatients	Cumulative incidence	Age, ethnicity, gender
Yawn, 2007^25^	USA	Retrospective passive surveillance study	1 January 1996–31 December 2001	Rochester Epidemiology Project, conducted in Olmsted County, Minnesota	≥50	ICD codes	Inpatients and outpatients	Incidence rate	Age, gender
Insinga, 2005^7^	USA	Retrospective passive surveillance study	1 July 2000–30 June 2001	Medstat MarketScan database	≥50	ICD codes	Inpatients and outpatients	Incidence rate	Age, gender, population (general population and IC only)
Mullooly, 2005^23^	USA	Retrospective passive surveillance study	1 January 1997–31 December 2002	Kaiser Permanente North-West California	≥45	ICD codes x estimated % of probable HZ	Inpatients and outpatients	Incidence rate	Age, gender, year
Yih, 2005^26^	USA	Retrospective active surveillance study	1 January 1999–31 December 2003	Behavioral Risk Factor Surveillance System survey, Massachusetts	≥45	Self-report	Inpatients and outpatients	Cumulative incidence	Age, year

Cumulative incidence: Number of new HZ cases per 1,000 population Incidence rate: Number of new HZ cases per 1,000 person-years GP, general practitioner/practice; GPRD, general practice research database; HZ, herpes zoster; IC, immunocompetent; ICD, International Classification of Diseases; ICPC, International Classification of Primary Care; NL, The Netherlands; NR, not reported; QAT, quality assessment tool; UK, United Kingdom; USA, United States of America.

Figure 2A provides the distribution of the 69 included studies by study design. Most studies (n = 51) were passive surveillance studies, with either a retrospective (n = 50) Table 1 or prospective (n = 1)^63^ design. Furthermore, 15 active surveillance studies (10 prospective [Table 1] and 5 retrospective^15,26,66–68^), and 3 unmatched retrospective cohort studies^21,24,34^ were found.

Figure 2B provides the distribution of the 69 included studies by the method used for HZ case identification. Fifty-two studies used the International Classification of Diseases (ICD) codes or equivalent codes to define HZ cases (Table 1), while 9 studies also used antiviral drug prescriptions (Table 1), 10 studies used a clinical diagnosis based on symptoms (Table 1), 5 studies used a self-report of HZ (Table 1), and one study used a natural language processing software algorithm (Table 1).

Figure 2C provides the distribution of the studies by the type of incidence data reported. More studies (n = 42; Table 1) expressed the HZ incidence as cumulative incidence than as incidence rate (n = 27; Table 1). While 6 studies gave only one overall HZ incidence (Table 1), 58 studies stratified the incidence by age (Table 1), 33 studies by gender (Table 1), 23 studies by study year (Table 1), and 4 studies by ethnicity.^15,17,21,38^ In 5 studies, the HZ incidence was reported for the overall general population as well as the immunocompetent population only.^6,21,24,41,71^ Lastly, 3 studies reported the HZ incidence for two different case definitions of HZ.^21,64,65^

Thirty-six of the included studies were nationwide studies or claimed to be representative of the national population, while the remaining studies were conducted in one or several country regions. Study periods varied, with the oldest data reported for the period 1980–1989^19^ and the most recent data for the year 2017.^49^ The number of studies including outpatients only (n = 32) was similar to the number of studies including both inpatients and outpatients (n = 37) (Table 1). The HZ incidence was reported for a study population ≥50 YOA in 42 studies (Table 1). In 12, 2 and 6 studies, the study population was ≥45 YOA (Table 1), ≥60 YOA^49,53^ and ≥65 YOA (Table 1), respectively. In the remaining studies, the HZ incidence was described for a study population of ≥70 YOA,^80^ >80 YOA,^60^ 60–89 YOA,^34^ 50–74 YOA,^45^ 50–64 YOA,^20^ 70–79 YOA,^46^ or 68–79 YOA.^39^

### Overview of the incidence of HZ

#### Overall incidence of HZ in the general population

**Figure 3.**
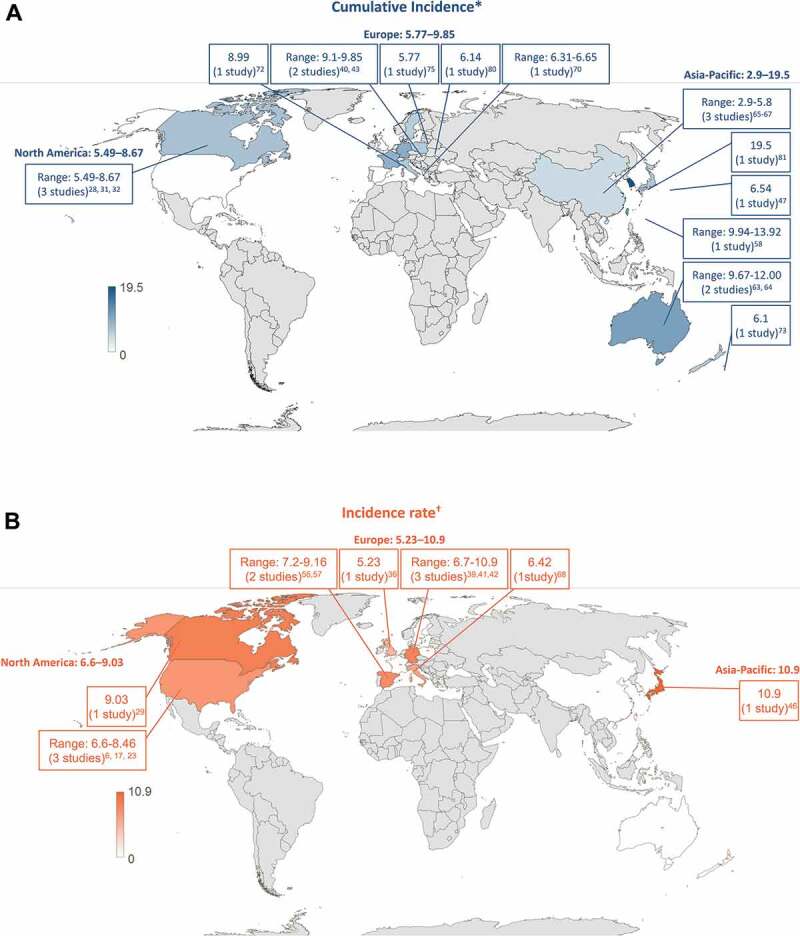
Overall HZ incidence in the general population ≥50 years by country (A) Cumulative incidence (B) Incidence rate

The overall HZ incidence in the general population ≥50 YOA was reported in 30 of 42 studies (Figure 3). When comparing the geographical regions, the cumulative incidence ranges were 5.49–8.67 per 1,000 population for North America, 5.77–9.85 per 1,000 population for Europe and 2.9–19.5 per 1,000 population in the Asia-Pacific region. Incidence rates were 6.6–9.03 per 1,000 person-years for North America, 5.23–10.9 per 1,000 person-years for Europe and 10.9 per 1,000 person-years in the Asia-Pacific region.

*Number of new HZ cases per 1,000 population † Number of new HZ cases per 1,000 person-yearsHZ, herpes zoster

#### Trends in cumulative incidence, over time

Figure 4A shows the cumulative incidence of HZ which is seen to be increasing over time. It should be noted that these studies do not all cover the same age groups, hence a wide difference in incidence estimates is observed. Most studies report the observed increase of HZ incidence over time. One study by Yih et al. specifically presented the relationship between varicella vaccination introduction and HZ incidence increase over time.^26^

#### Incidence of HZ in the general population, by gender

The HZ incidence in the general population ≥50 YOA stratified by gender was reported in 14 studies. All 14 studies reported a higher incidence of HZ in females compared to males. In females, the cumulative incidence in the general population ≥50 YOA ranged from 3.22 cases per 1,000 population in 2010 in China^66^ to 11.2 cases per 1,000 population in 2004 in Germany.^41^ In males, the corresponding incidence ranged from 2.44 per 1,000 persons to 8.0 per 1,000 persons in the same studies.^41,66^ The incidence rate in females ranged from 6.05 cases per 1,000 person-years in the period 2006–2012 in the UK^37^ to 12.8 cases per 1,000 person-years in the period 2008–2012 in Japan.^47^ In males, the corresponding incidence ranged from 4.30 cases per 1,000 person-years to 8.5 cases per 1,000 person-years in the same studies.^37,47^

Twenty-eight studies reported a higher incidence of HZ in females than for in males in age groups besides the ≥50 years group. However, Chaves et al. reported a higher HZ incidence in males in the ≥65 years group, but corresponding confidence intervals were wide and overlapped.^15^ In two studies a higher incidence in males was found in the highest age groups only.^23,74^

#### Incidence of HZ in the general population, by age

Almost all studies (n = 58) reported the HZ incidence for different age groups (Supplementary Table 4). In 35 studies, the incidence increased with increasing age. However, in 14 studies a drop in incidence was reported for the highest age groups, i.e., ≥70 YOA,^67,82^ ≥75 YOA,^56^ ≥80 YOA,^42,47,48,57,61,71^ or ≥85 YOA.^37,62,69,75,79^ In 8 studies, both scenarios (i.e. increasing incidence with increasing age and a decline in incidence for the highest age group) were reported during different time periods.^17,23,31–33,55,59,64^ Moreover, in one study the incidence of HZ decreased in individuals of 60–69 YOA compared to those of 50–59 YOA, after which it increased in individuals ≥70 YOA.^54^ Except for one Japanese study that did not report the incidence for persons ≥75 YOA^45^ and one Chinese study,^66^ all Asian studies reported a drop in incidence for the highest age groups.^47,48,59,61,62,67,82^ Furthermore, 2 Italian studies,^69,71^ 2 Spanish studies,^56,57^ one UK study^37^ and one study from New Zealand^75^ reported a decline in the incidence for the highest age groups. From the other European countries, Australia, Canada and the USA, no studies were found that solely reported a declining incidence in the highest age groups.

#### Trends in cumulative incidence, by age

The cumulative incidence for all geographical regions by age is provided in Figure 4B (data for all individual studies is provided in Supplementary Figure 1). Most studies depict a steady increasing trend in HZ incidence with age. Four studies covering North America,^15^ Europe^70^ and two studies from the Asia-Pacific region^68,82^ report incidence estimates that deviate from the overall trend.^15,68,70,82^

#### Incidence of HZ in immunocompetent persons

Nine studies reported the HZ incidence in immunocompetent persons only.^6,21,24,37,39,41,58,71,73^ In seven studies, the HZ incidence for those ≥50 YOA was reported, with a range of 6.31–9.5 cases per 1,000 persons^41,71,73^ and a range of 5.23–7.2 cases per 1,000 person-years.^6,24,37,58^ In five studies, the HZ incidence was separately reported for the overall general population and the immunocompetent population only.^6,21,24,41,71^ In all five studies, estimates of incidence in the overall general population were numerically higher compared to the immunocompetent population.

#### Incidence of HZ in the general population, by ethnicity

In 4 studies, the HZ incidence was reported by ethnicity.^15,17,21,38^ In 3 studies, the highest incidence was reported in Caucasians, while in the fourth study, persons with an American Indian/Alaskan native ethnicity were found to have the highest HZ incidence.^17^ The lowest HZ incidence was generally found among persons reported as black, except in the study of Chaves et al. where those with either Hispanic or other ethnicity had the lowest HZ incidence.^15^

#### Incidence of HZ in the general population, by study year

In 23 studies the HZ incidence was reported for different years, and in 17 of these studies the HZ incidence increased during the years. In addition, Amirthalingam et al. also reported such an increase until the introduction of the zoster vaccine in the UK.^34^ Harpaz et al. also found an increase in HZ incidence over time, but specifically reported a decline in the rate of increase among older adults from 2006 through 2016; reasons for this trajectory over time for older adults could not be confirmed as a consequence of the impact of HZ vaccination introduction.^27^ In the remaining five studies, no clear increasing trend over the years was reported.^20,32,33,57,68^

#### Incidence of hz in the general population, by case definition

In 3 studies, the HZ incidence was compared for different case definitions of HZ. Langan et al. reported the HZ incidence based on ICD codes only and based on these ICD codes in combination with the use of antivirals within 7 days before or after the diagnostic code for HZ.^21^ Among those individuals ≥65 YOA, the incidence was much lower when using the latter definition (9.9 per 1,000 person-years) compared to ICD codes only (15.0 per 1,000 person-years). The lower incidence using the latter definition remained when the data were stratified for different age, gender and ethnicity groups. Two Australian studies compared the HZ incidence based on general practitioner (GP) visits with incidence estimates based on antiviral prescriptions. In both studies, no clear difference was found between both case definitions.^64,65^

### Quality assessment of the included studies

The methodological quality of each publication was assessed using the quality assessment tool provided in Supplementary Table 3. The majority of studies had a valid case definition for the diagnosis of HZ and the denominator to calculate incidence was properly defined. However, the majority of studies did not capture individuals that were representative of the target population (Supplementary Table 5).

## Discussion

This review provides an overview of the worldwide incidence of HZ in the general population with data from 69 studies. It also provides important contemporary insights on the incidence of HZ by gender, age, immunocompetent status, ethnicity, study year and case definition since the last published systematic review on this topic by Kawai et al.^4^ in 2014. It should be noted that this review found little to no evidence for the regions of Eastern Europe, Middle East, South America or Africa. HZ may be a low health priority in many of these countries; however, the proportion of older adults is projected to double over the next several decades,^14^ and the numbers of HZ cases may increase worldwide. The evidence base from Kawai et al.^4^ together with this review provides a comprehensive overview of how an appropriate study methodology leads to consistent methods being used for worldwide incidence studies to help deliver reliable estimates of disease burden. These updated data could also be utilized in the context of healthcare policy surrounding the implementation of effective preventive measures such as vaccination against HZ.

Overall, the cumulative incidence of HZ ranged from 2.9 to 19.5 cases per 1,000 population and from 5.23 to 10.9 cases per 1,000 person-years in the general population ≥50 YOA. Among geographical regions, the highest and lowest incidence rates were both reported in the Asia-Pacific countries, although incidence estimates overlap, and the lowest incidence came from the same region. In the general population ≥50 YOA, the cumulative incidence (3.22–11.2 versus 2.44–8.0 cases per 1,000 population) and incidence rates (6.05–12.8 versus 4.30–8.5 cases per 1,000 person-years) were higher in females than in males. The incidence of HZ was higher in Caucasians compared to persons of Black ethnicity, but this was examined in only a few studies and could be attributed to under-reporting. Only a few studies reported incidence data based on these stratifications. Across regions, a trend of steadily increasing HZ incidence over time is observed.

Variations in incidence estimates summarized in this review could be related to several factors and warrant discussion in the interpretation of the overall review findings. Methodological variations related to geographical spread, sample size, diagnostic methodology, time period and age of the study population were observed among studies. This made comparisons between studies difficult while simultaneously affecting generalizability. Specifically, studies varied in terms of study setting (ranging from a single region to national surveillance), and sample size (ranging from 2,135 to 31,943,930 individuals) with information on sample size lacking in 32 studies. In some studies, antiviral prescriptions were used to define HZ. Individuals with a mild case of HZ may not receive formal treatment but may choose to obtain over-the-counter treatment; it is also expected that treatment uptake differs by age which could have led to differential case ascertainment. These factors could have led to an underestimation of the overall HZ incidence. To the contrary, the overall HZ incidence may have been overestimated if only antivirals were used to confirm HZ diagnosis, attributable to the fact that antivirals are also prescribed for other diseases. Additionally, the study period also differed between publications, with the oldest data reporting for the period 1980–1989 and the most recent data for the year 2017. As HZ incidence tends to increase over the years and differs by age, the study period and the age of the study population should be kept in mind when comparing studies.

Most studies revealed a trend of increasing incidence of HZ with increasing age. A few deviations were seen on this trend in different regions, where the highest age groups (i.e. >80 YOA) reported a drop in incidence. Reasons for this observation could be that older adults have pain and rash due to other conditions and, as such, HZ may be under-diagnosed, or the fact that many subjects in insurance databases represent a healthier cohort of individuals.^45^ In addition, GP visits for individuals who are institutionalized or in nursing homes are not always captured in traditional databases. In this review, half of the studies included outpatient-only settings, which lends bias toward the representation of subjects with milder disease (i.e. hospitalization not required). It is well documented that the risk of hospitalization increases with age,^9^ thereby consequently under-representing the incidence in older adults. Other studies can be considered as outliers when looking at the overall trend with age, as they report either lower or higher incidences. The deviations could generally be explained by the type of study design, choice of population, case definition and healthcare-seeking behavior of individuals in a specific setting. Some of these studies used different case definitions, for example; self-reporting using a random digit dialing survey;^15^ use of clinical confirmation only via GP clinics;^70^ and self-reporting via surveying individuals door to door^68^ and a national database study that captures mild cases with an over-representation of females^82^).

The results of the quality assessment of the included studies revealed that a majority of the 69 studies had a valid case definition for the diagnosis of HZ and the denominator to calculate incidence was generally properly defined. However, most studies did not capture individuals that were representative of the national population. These methodological aspects are seldom reported in the individual studies yet useful in contextualizing and interpreting data from epidemiological studies. In this review, it was often unclear from the individual studies whether these regional studies were representative of the overall national population due to several reasons. First, 36 of the included studies were nationwide studies or claimed to be representative of the national population, while the remaining studies were conducted in one or several regions. Second, in all studies, baseline characteristics of the older adults were generally lacking, as the focus of most of the studies was on the total population instead of older adults. Third, most of the included studies in this review were passive surveillance studies that utilized a retrospective design which could have issues surrounding quality of data or incomplete datasets. Information coming from these databases is often dependent on the quality of the information reported by the physicians, with the possibility of mis-coding or under-diagnosis. Fourth, few of these studies reported using a validated algorithm to detect HZ while many of the studies noted misclassification of HZ as a potential limitation. Many of the active surveillance studies failed to provide the proportion of eligible patients that were finally enrolled, making it difficult to state whether the results were generalizable to the overall population. In some of these studies a HZ case was defined by self-reporting, the accuracy of which was never verified, as self-report of HZ is subject to recall bias. Additionally, misclassification of HZ disease due to other rashes (e.g., herpes simplex) or other rashes classified as HZ based on ICD codes may also have occurred. Thus, misclassification of HZ could inadvertently lead to an underestimation or overestimation of HZ cases in the overall population.

### Review limitations

Several limitations of this review are worth noting in the interpretation of the overall findings. The search strategy was not designed to find publications on the epidemiology or disease burden of HZ in general. Therefore, some publications that did not specify incidence in the title or abstract or to which an incidence medical subject heading term was not assigned, may not have been captured in this review. However, by screening all systematic reviews and meta-analyses for potentially relevant publications, we feel that this limitation has largely been overcome. A time limit was applied to the searches to identify publications beginning from 1 January 2002. This was considered appropriate by the authors for the update of the evidence base on incidence as a previous review by Kawai et al. summarizes the incidence data from studies that covers time periods from as early as 1945 until 2012.^4^ Another limitation of this review is the use of an invalidated checklist to perform a quality check of the included studies. However, the quality check performed in this review is simplistic but sheds light on important gaps in the study methods and results reported in the individual studies. Performing such quality checks could drive improvement and harmonization of reporting standards for these types of publications in the future.

## Conclusion

Over the last few decades, the incidence of HZ has increased with increasing age due to the aging of the population worldwide, and this trend is visible independent of geographic location.^10^ Independently of geographic location, the world’s population is aging: the number of older persons is rising, and older age groups constitute a growing share of the population in nearly every country, with implications foreseen for the healthcare sector among others. The aim of many healthcare systems around the world is to focus on promoting healthy aging to prevent diseases and chronic conditions. In this context, the occurrence of HZ, and its associated complications, is expected to place an additional burden, especially in older patients who already have health problems to cope with in their everyday life. Effective vaccines to prevent HZ are available and are known to have a substantial positive impact on improving the quality of life and on decreasing the burden of complications associated with HZ in older individuals.

**Figure 4.**
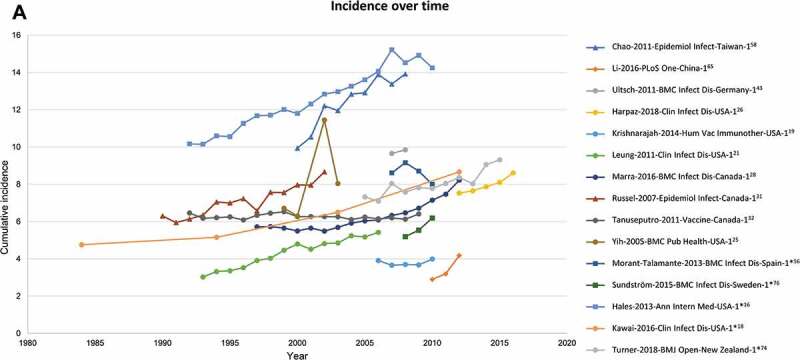
Cumulative HZ incidence (number of new HZ cases per 1,000 population) by (A) Time, (B) Age (region).Source: Table 1Figure 2A: Studies with at least 2 years of data are depicted*Incidence rate (number of HZ cases per 1,000 person-years) converted to cumulative incidence (number of HZ cases per 1,000 population)HZ, herpes zosterNote: While 42 publications reported cumulative incidence, only 30 of these presented an overall incidence for those ≥50 years and are depicted here

**Figure 4.**
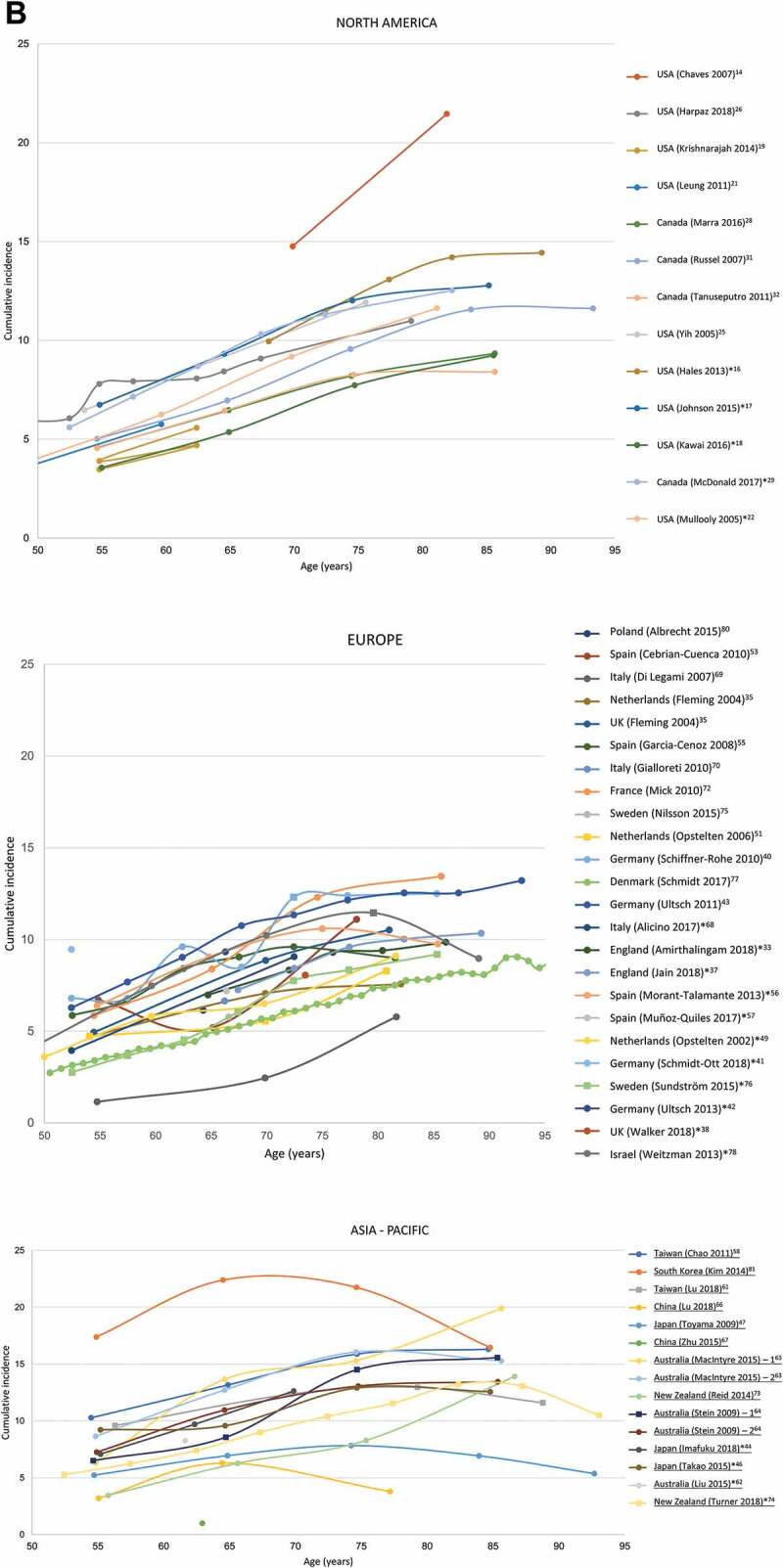
(Continued)

## Supplementary Material

Supplemental Material

## Plain language summary

**What is the context?**

- Herpes Zoster (HZ) occurs due to the reactivation of the varicella zoster virus and is common among adults ≥50 years of age (YOA) as cell-mediated immunity declines due to aging.
- Age is a significant risk-factor related to HZ and an increase in life expectancy of the general population may considerably increase the number of HZ cases.
- In the context of the world’s aging population, the occurrence of HZ is expected to place an additional burden on patients ≥50 YOA who might already have other health problems to cope with in their everyday life.

**What is new?**

- This systematic review of 69 full-text publications demonstrates that the worldwide incidence of HZ is high among individuals ≥50 YOA with little variation in the different regions of the world.
- When comparing the geographical regions, the highest incidences were mostly reported in the Asia-Pacific countries.
- Our results suggest that the incidence of HZ is higher in females than in males.
- The majority of studies also reported a higher incidence of HZ with increasing age.
- Incidence rates did not seem to differ when different case definitions were used.
- By ethnicity, incidence rates were the highest among Caucasians and the lowest among Black, Hispanic or other ethnicities.
- Studies which reported HZ incidence by year mostly showed an increase in incidence over time.

**What is the impact?**

- This review provides a comprehensive overview of HZ disease burden, that could be considered during the planning and implementation of preventive measures such as vaccination against HZ.
- Health care systems around the world are increasingly focusing on promoting healthy aging to prevent diseases and chronic conditions. In this context, the prevention of HZ among the ≥50 YOA population could bring about a reduction in the risks to their health and improve their quality of life.
