# Supplementary material for: A systematic literature review of herpes zoster incidence worldwide
Source: Hum Vaccin Immunother. 2021 Mar 2;17(6):1714–32. doi: 10.1080/21645515.2020.1847582 (PMC8115759; doi:10.1080/21645515.2020.1847582)
Supplement: Supplemental Material [file KHVI_A_1847582_SM1670.pdf]

SUPPLEMENTARY MATERIAL

Supplementary Table 1: Search strategy

**Database**	**Strategy**	**No. of publications**
Medline (via PubMed)	*#1 Herpes Zoster*<br><br>"Herpes Zoster"[Mesh] OR zoster[tiab] OR shingle*[ti,ab]<br><br>*#2 Incidence*<br><br>"Incidence"[Mesh] OR inciden*[ti,ab]<br><br>Limits<br>Publication date from 01-01-2002.	1,349
Embase	*#1 Herpes Zoster*<br>'Herpes Zoster'/exp OR zoster:ti,ab OR shingle*:ti,ab<br><br>*#2 Incidence*<br>'incidence'/exp OR inciden*:ti,ab<br><br>Limits<br>Publication date from 01-01-2002.<br><br>Publication type: article, article in press, review (this is applied in Embase to avoid a lot of articles of non-pertinent type, such as conference abstracts)	2,128
VHL	*#1 Herpes Zoster*<br>(mh:(herpes zoster)) OR (ti:(zoster)) OR (ab:(zoster)) OR (ti:(shingle*)) OR (ab:(shingle*))<br><br>*#2 Incidence*<br>(mh:(incidence)) OR (ti:(inciden*)) OR (ab:(inciden*))<br><br>Limits<br>Publication date from 01-01-2002.	1,371

Supplementary Table 2: Inclusion and exclusion criteria

	Inclusion criteria	Exclusion criteria
Population	• Studies in persons ≥50 YOA	• All other age groups<br>• Non-representative population (e.g. military personnel)
Intervention	• Not limited by intervention	• NA
Comparator	• Not limited by comparator	• NA
Outcome	• Incidence of HZ	• HZ incidence in patients with another condition (e.g. immunocompromised persons). In case a study compared the incidence in a population with and without a certain condition, the full-text was checked to see whether an overall incidence could be calculated for both groups combined.<br>• All other topics besides incidence
Study design	• Primary peer-reviewed research<br>○ Observational studies (e.g. cohort studies, case-control studies, pre-/post-vaccine introduction time series)<br>○ Surveillance studies	• Non-primary research<br>○ Systematic reviews*<br>○ Meta-analyses*<br>○ Narrative reviews (without methods)<br>○ Public health programs or recommendations<br>○ Transmission modeling studies<br>○ Cost-effectiveness or health economics studies<br>• Case reports<br>• Letter to editor

		• Newspaper<br>• Editorial<br>• Comment<br>• Opinion paper<br>• Phase II/III trials<br>• Molecular studies<br>• Studies with insufficient methodological details
**Limits**		
Publication date	January 2002 – December 2018	Articles published outside of dates considered eligible for inclusion
Geographic scope	Worldwide	None
Language	English, German, Dutch, French, Spanish and Italian	Articles published in any other language besides those considered eligible for inclusion

HZ, herpes zoster; NA, not applicable; YOA, years of age.

*References of included articles in these systematic reviews/meta-analyses were manually screened for additional relevant original articles (as deemed necessary by the reviewer).

Supplementary Table 3: Quality assessment tool

Q1. Are the individuals captured in the study setting likely to be representative of the target population?

- *Yes: Study is representative of a broad population (e.g. regional vs. country level, age groups, health care setting, socio-demographic status).*

  Representative healthcare setting:
  - *General practice-based records*
  - *Health insurance database*
- *No: Study sample is not representative of a broad population. No representative healthcare setting:*
  - *Hospital-based records*
  - *Population-based screening*
  - *Pharmacy database*
  - *Reports from clinical practice* (this is when they just report what they think they remember seeing but do not get all cases from general practice-based records)

Q2. Is there a valid case definition for the diagnosis of herpes zosterHZ patients?

- *Yes: A valid method was used to define a case of HZ within the study.*
  - *ICD codes with chart/laboratory review confirmation*
  - *ICD codes only*
  - *Clinical diagnosis but with PCR confirmation*

- *No: Unclear or no valid method was used to define HZ.*
    - *ICD codes AND antiviral prescriptions*
    - *ICD codes OR antiviral prescriptions*
    - *Antiviral prescriptions only*
    - *Self-reported diagnosis*
    - *Clinical diagnosis, without stating this comes from medical records*
    - *No clear description of case definition*

Q3. Is the denominator properly defined to calculate HZ incidence?

- *Yes: Denominator to calculate HZ incidence (per person-time at risk or per study population at risk) calculated appropriately.*
- *No: Denominator either not clearly defined or calculated inaccurately.*

ICD, International Classification of Diseases; HZ, herpes zoster; PCR, polymerase chain reaction.

Supplementary Table 4: HZ incidence in the general population by age

Age group	Cumulative incidence (no. of HZ cases per 1,000 population) Range (no. of studies)	Incidence rate (no. of HZ cases per 1,000 person-years) Range (no. of studies)
≥45 years	2.54 (1 study)[69]	8.2 (1 study)[62]
45-54 years	2.36-6.56 (3 studies)[21, 26, 71]	3.6-4.44 (2 studies)[49, 78]
50-54 years	3.79-6.9 (6 studies)[30, 40, 43, 55, 70, 75]	2.5-6.2 (6 studies)[29, 36, 41, 68, 74, 76]
55-59 years	4.56-7.76 (6 studies)[30, 40, 43, 55, 70, 75]	3.2-7.46 (6 studies)[29, 36, 41, 68, 74, 76]
50-59 years	1.72-17.4 (15 studies)[26, 28, 31, 32, 47, 53, 58, 60, 63-66, 72, 73, 81]	2.44-6.07 (11 studies)[6, 15, 17, 18, 22-24, 42, 44, 46, 56]
≥60 years	6.18-16.21 (4 studies)[48, 58, 63, 73]	7.6-10.46 (2 studies)[17, 33]
45-64 years	1.15-7.42 (4 studies)[25, 34, 54, 69]	-
50-64 years	3.55-9.98 (2 studies)[19, 61]	-
55-64 years	4.09-8.29 (3 studies)[21, 26, 71]	5.8-7.48 (2 studies)[49, 78]
60-64 years	4.12-9.6 (8 studies)[19, 30, 40, 43, 52, 55, 70, 75]	3.9-9.71 (8 studies)[23, 29, 36, 41, 44, 68, 74, 76]
≥65 years	3.98-20.03 (9 studies)[14, 21, 25-27, 34, 50, 52, 69]	7.89-15.23 (6 studies)[15, 16, 20, 23, 37, 78]
65-69 years	6.1-10.85 (6 studies)[30, 40, 43, 55, 70, 75]	5.5-12.73 (9 studies)[16, 20, 29, 36, 37, 41, 68, 74, 76]
60-69 years	2.43-22.4 (15 studies)[26, 28, 31, 32, 47, 53, 58, 60, 63-66, 72, 73, 81]	4.62-9.41 (10 studies)[6, 15, 17, 18, 22, 24, 33, 42, 46, 56]
≥70 years	3.8-11.77 (5 studies)[26, 53, 60, 66, 79]	-
65-74 years	2.45-15 (5 studies)[14, 54, 61, 69, 71]	6.5-12.63 (3 studies)[44, 49, 78]
70-74 years	7.12-12.3 (6 studies)[30, 40, 43, 55, 70, 75]	6.34-14.2 (10 studies)[16, 20, 29, 33, 36, 37, 41, 68, 74, 76]
≥75 years	5.78-25 (4 studies)[14, 54, 55, 69]	9.1-12.97 (2 studies)[29, 49]
75-79 years	7.28-12.4 (5 studies)[30, 40, 43, 70, 75]	7.09-15.95 (9 studies)[16, 20, 33, 36, 37, 41, 68, 74, 76]
70-79 years	3.2-21.8 (13 studies)[28, 31, 32, 45, 47, 58, 60, 63-65, 72, 73, 81]	7.13-12.12 (9 studies)[6, 17, 18, 22, 24, 38, 42, 46, 56]
≥80 years	6.09-19.89 (11 studies)[28, 32, 40, 58, 60, 63-65, 72, 73, 81]	7.95-13.67 (13 studies)[6, 17, 18, 20, 22, 24, 41, 42, 46, 56, 59, 62, 76]
75-84 years	9.85-13.52 (2 studies)[61, 71]	9.9-11.45 (2 studies)[33, 78]
80-84 years	7.67 -12.65 (4 studies)[30, 43, 70, 75]	7.29-18.5 (5 studies)[16, 36, 37, 68, 74]
85-89 years	12.05-13.23 (2 studies)[30, 43]	9.0-18.4 (1 study)[74]
80-89 years	6.93-13.76 (2 studies)[31, 47]	9.9-12.8 (2 studies)[33, 74]
≥85 years	5.85-12.41 (3 studies)[61, 70, 75]	5.59-16.90 (5 studies)[16, 36, 37, 68, 78]
≥90 years	5.37-15.27 (4 studies)[30, 31, 43, 47]	6.8-12.9 (1 study)[74]
85-94 years	10.77 (1 study)[71]	-
90-94 years	-	11.9 (1 study)[74]

≥95 years	9.45-14.37 (2 studies)[71, 77]	4.7 (1 study)[74]
Per year from 50 years until 94 years	2.15 (50 years) – 8.44 years (94 years) (1 study)[77]	-

HZ, herpes zoster

Supplementary Table 5: Quality assessment results (N=69)

Reference	Are the individuals captured in the study setting likely to be representative of the target population?	Is there a valid case definition for the diagnosis of herpes zoster patients?	Is the denominator properly defined to calculate HZ incidence?
Albrecht, 2015[80]	No	Yes	Yes
Alicino, 2017[68]	No	No	No
Amirthalingam, 2018[33]	Yes	Yes	Yes
Brisson, 2003[34]	No	No	Yes
Cebrian-Cuenca, 2010[53]	No	No	Yes
Chao, 2011[58]	Yes	Yes	Yes
Chaves, 2007[14]	No	No	Yes
Chen, 2014[15]	Yes	Yes	Yes
Di Legami, 2007[69]	No	No	Yes
Edgar, 2007[27]	No	Yes	Yes
Esteban-Vasallo, 2014[54]	No	Yes	Yes
Fleming, 2004[35]	No	No	Yes
Garcia-Cenoz, 2008[55]	No	Yes	Yes
Gauthier, 2009[36]	Yes	Yes	Yes
Gialloreti, 2010[70]	No	Yes	Yes
Gonzalez Chiappe, 2010[71]	No	No	Yes
Hales, 2013[16]	Yes	Yes	Yes
Harpaz, 2018[26]	Yes	Yes	Yes
Hillebrand, 2015[39]	Yes	Yes	Yes
Imafuku, 2018[44]	Yes	No	Yes
Insinga, 2005[6]	Yes	Yes	Yes
Jain, 2018[37]	Yes	Yes	Yes

Jih, 2009[59]	Yes	Yes	Yes
Johnson, 2015[17]	Yes	Yes	Yes
Kawai, 2016[18]	No	Yes	No
Kim, 2014[81]	Yes	Yes	Yes
Krishnarajah, 2014[19]	Yes	Yes	Yes
Langan, 2013[20]	Yes	Yes*	Yes
Leung, 2011[21]	Yes	Yes	Yes
Li, 2016[65]	No	No	No
Lin. 2010[60]	Yes	Yes	Yes
Liu. 2015[62]	No	No	Yes
Lu, 2018[61]	Yes	Yes	Yes
Lu, 2018[66]	No	No	Yes
MacIntyre, 2015[63]	Yes**	Yes**	Yes
MacIntyre-PBS 2015	No	No	Yes
Marra, 2016[28]	No	Yes	Yes
McDonald, 2017[29]	No	Yes	Yes
Mick, 2010[72]	No	Yes	Yes
Morant-Talamante, 2013[56]	No	Yes	Yes
Mullooly, 2005[22]	No	Yes	No
Muñoz-Quiles, 2017[57]	No	Yes	Yes
Nilsson, 2015[75]	No	No	Yes
Opstelten, 2002[49]	Yes	Yes	No
Opstelten, 2005[50]	Yes	Yes	Yes
Opstelten, 2006[51]	Yes	Yes	Yes
Pierik, 2012[52]	No	Yes	Yes
Reid, 2014[73]	No	No	Yes
Rimseliene, 2016[79]	Yes	Yes	Yes
Russel, 2007[31]	No	Yes	Yes

Russel, 2014[30]	No	Yes	Yes
Schiffner-Rohe, 2010[40]	No	Yes	Yes
Schmidt, 2017[77]	No	No	Yes
Schmidt-Ott, 2018[41]	No	No	No
Shiraki. 2017[45]	No	Yes	Yes
Stein, 2009[64]	Yes	Yes	Yes
Stein-PBS 2009	No**	No**	Yes
Suaya, 2014[23]	Yes	Yes	Yes
Sundström, 2015[76]	No	Yes	No
Takao, 2015[46]	No	Yes	Yes
Tanuseputro, 2011[32]	No	Yes	Yes
Toyama, 2018[48]	No	No	Yes
Toyama, 2009[47]	No	No	Yes
Turner, 2018[74]	No	No	No
Ultsch, 2011[43]	Yes	Yes	Yes
Ultsch. 2013[42]	No	Yes	Yes
Walker, 2018[38]	Yes	Yes	Yes
Weitzman, 2013[78]	Yes	Yes	No
Yawn, 2007[24]	No	Yes	No
Yih, 2005[25]	No	No	Yes
Zhu, 2015[67]	No	No	No

* For the other case definition based on drug prescription, this entry was assigned as “No”.

** For the dataset (based on PBS), this entry was assigned as “No”.

Supplementary Figure 1: Cumulative HZ incidence from individual studies (N=69)

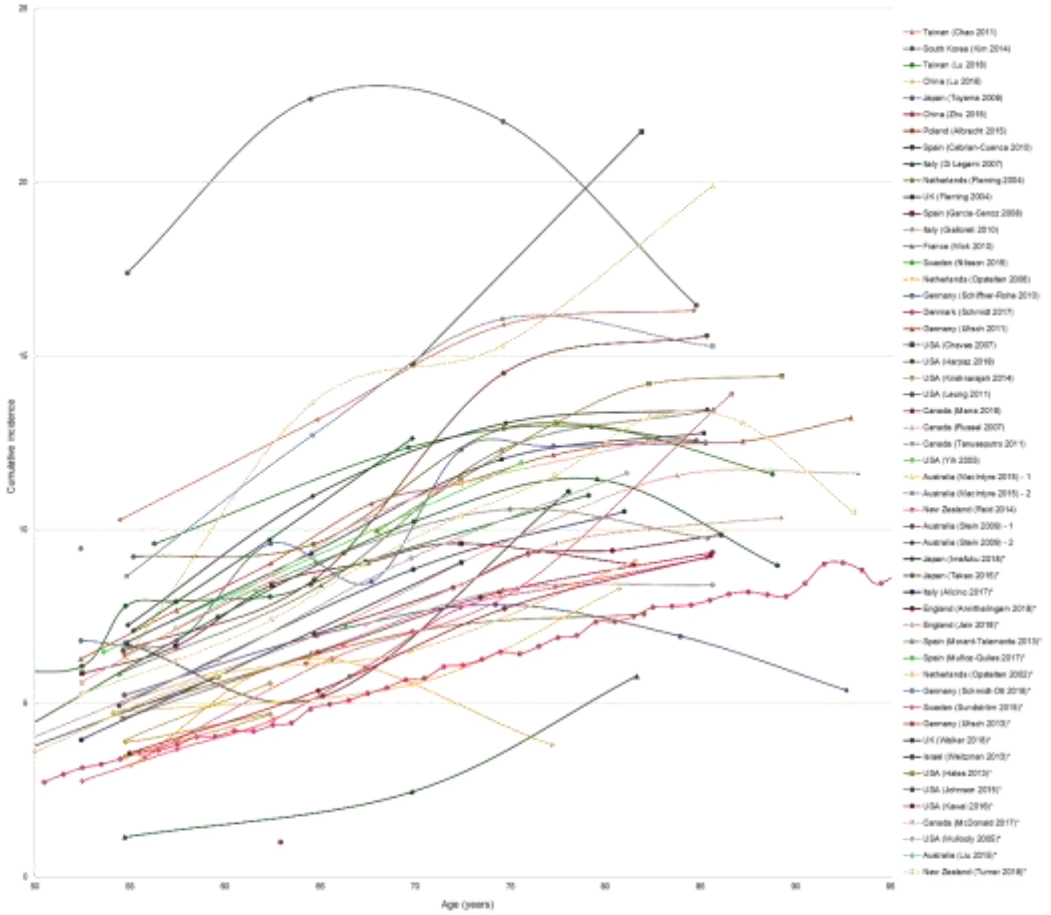

*Incidence rate (number of HZ cases per 1,000 person-years) converted to cumulative incidence (number of HZ cases per 1,000 population)
HZ, herpes zoster
